# The bZIP Transcription Factor HAC-1 Is Involved in the Unfolded Protein Response and Is Necessary for Growth on Cellulose in *Neurospora crassa*

**DOI:** 10.1371/journal.pone.0131415

**Published:** 2015-07-01

**Authors:** Alejandro Montenegro-Montero, Alejandra Goity, Luis F. Larrondo

**Affiliations:** Millennium Nucleus for Fungal Integrative and Synthetic Biology, Departamento de Genética Molecular y Microbiología, Facultad de Ciencias Biológicas, Pontificia Universidad Católica de Chile, Santiago, Chile; University of California, UNITED STATES

## Abstract

High protein secretion capacity in filamentous fungi requires an extremely efficient system for protein synthesis, folding and transport. When the folding capacity of the endoplasmic reticulum (ER) is exceeded, a pathway known as the unfolded protein response (UPR) is triggered, allowing cells to mitigate and cope with this stress. In yeast, this pathway relies on the transcription factor Hac1, which mediates the up-regulation of several genes required under these stressful conditions. In this work, we identified and characterized the ortholog of the yeast *HAC1* gene in the filamentous fungus *Neurospora crassa*. We show that its mRNA undergoes an ER stress-dependent splicing reaction, which in *N*. *crassa* removes a 23 nt intron and leads to a change in the open reading frame. By disrupting the *N*. *crassa hac-1* gene, we determined it to be crucial for activating UPR and for proper growth in the presence of ER stress-inducing chemical agents. Neurospora is naturally found growing on dead plant material, composed primarily by lignocellulose, and is a model organism for the study of plant cell wall deconstruction. Notably, we found that growth on cellulose, a substrate that requires secretion of numerous enzymes, imposes major demands on ER function and is dramatically impaired in the absence of *hac-1*, thus broadening the range of physiological functions of the UPR in filamentous fungi. Growth on hemicellulose however, another carbon source that necessitates the secretion of various enzymes for its deconstruction, is not impaired in the mutant nor is the amount of proteins secreted on this substrate, suggesting that secretion, as a whole, is unaltered in the absence of *hac-1*. The characterization of this signaling pathway in *N*. *crassa* will help in the study of plant cell wall deconstruction by fungi and its manipulation may result in important industrial biotechnological applications.

## Introduction

The endoplasmic reticulum (ER) is crucial for the production of membrane and secreted proteins and consequently, its function is under tight control. To maintain protein folding homeostasis in the ER, the cell must balance the ER protein folding capabilities to the protein flux through the secretory pathway. When protein folding requirements exceed the ER’s folding capabilities, unfolded proteins accumulate within this organelle, a condition known as ER stress. ER resident transmembrane sensors then trigger a conserved signaling pathway known as the unfolded protein response (UPR) (reviewed in [1, 2]). Activation of these sensors lead to a major transcriptional program aimed at increasing folding capacity in the ER and adjusting the secretory pathway [3], while also mediating a decrease in ER protein load through selective mRNA degradation and translational repression [4–7], as well as a global reduction in protein synthesis [8]. These mechanisms, along with others [9], work together to revert ER stress and re-attain protein folding homeostasis in the ER [10].

In eukaryotic microorganisms such as budding yeast, where this pathway has been most extensively studied, the only identified ER stress sensor is Ire1, an ER-resident transmembrane protein that has kinase and endoribonuclease activity [11, 12]. The expression of UPR target genes is controlled by a key member of this regulatory branch, the bZIP transcription factor Hac1 [13, 14]. Upon sensing unfolded proteins on its luminal side, Ire1 oligomerizes, *trans*-autophosphorylates and undergoes conformational changes that ultimately lead to the activation of its cytosolic RNase domain (reviewed in [2, 15]). Once activated, this domain specifically cleaves its only known target, the *HAC1* mRNA, in two sites, releasing a 252 nt intron, whereas the two resulting exons are then ligated together by the tRNA ligase Trl1/Rgl1 (reviewed in [1, 2]. This unconventional splicing reaction relieves the transcript from translational repression and leads to the generation of a potent transcriptional activator [2]. The Hac1 protein can then translocate into the nucleus and mediate a transcriptional program that ultimately affects over 5% of the *Saccharomyces cerevisiae* transcriptome, modulating multiple ER and secretory pathway functions, with the goal of alleviating ER stress [3]. Attenuation of the UPR is partially achieved through inactivation of Ire1 by the Ptc2 phosphatase [16]. In addition, in all fungal species evaluated so far, *hac1* or *ire1* deletions result in increased sensitivity to cell wall perturbing agents, suggesting a coordination between the responses to these related stress conditions [17].

Filamentous fungi have been exploited industrially for the production of economically relevant proteins due to their efficient, high-capacity protein secretion capabilities [18, 19]. The UPR has been studied in a limited number of these organisms mostly with the goal of overcoming bottlenecks encountered in the industrial production of these proteins [20, 21]. In this context, the UPR has been examined in commercially relevant filamentous fungi, including *Trichoderma reesei*, *Aspergillus nidulans*, and *Aspergillus niger* [22–26]. In general, the basic elements of the response are similar to those in budding yeast, with filamentous fungi exhibiting the widely conserved Ire1/Hac1 branch and regulating several aspects of ER function upon ER stress [22, 26]. One interesting difference is that the regulatory intron removed from the transcript of the *Hac1* homologs in these organisms is small (20 nt long), closer to the size described in plants and animals [27]. In addition, the 5’ end of the *Hac1* transcript has been reported to be truncated upon ER stress in these few evaluated filamentous fungi [22, 23, 28], another difference with the *S*. *cerevisiae* system. These findings highlight the differences that a particular (and even well characterized) process can have among different fungal species, supporting the need to further characterize it in different systems to address biodiversity and niche-associated peculiarities. Indeed, further differences can be found within fungi (this study and [24, 29]). Fungal UPR studies have gathered momentum not only due to the importance of protein secretion in industrially relevant fungi, but more recently because the UPR has been found to impact fungal lifestyle, particularly virulence, in both animal [28–31] and plant [32–34] pathogens. Whether other aspects of filamentous fungi lifestyles require a functional UPR, is largely unexplored.

Fungi are known to play an important role in the decomposition and recycling of organic material in their natural settings. To efficiently metabolize the complex polymeric substrates encountered in the wild, fungi exhibit exquisitely regulated secretion of numerous enzymes, a result of their saprobic lifestyles. Filamentous fungi such as *T*. *reesei*, *A*. *niger* and more recently *Neurospora crassa*, have been studied to dissect the molecular basis of plant cell deconstruction (reviewed in [35]). Importantly, the numerous molecular, genetic and biochemical tools available for *N*. *crassa* [36] have recently made this fungus an attractive system in which to study these processes and have propelled it as a platform for research in biofuel production [37].

In this work, we set out to characterize HAC1 and the unfolded protein response in *N*. *crassa*, which surprisingly, despite decades of cell biology research in this organism and the existence of a fully annotated genome [38], has not yet been studied. We show that the *N*. *crassa hac-1* mRNA undergoes an ER stress-dependent splicing reaction, which removes a 23 nt intron which changes the open reading frame. In addition, we establish that the *N*. *crassa* gene can complement a *S*. *cerevisiae HAC1* mutant. Disruption of *hac-1* in *N*. *crassa* reveals it to be essential for growth under ER stress and for activating the UPR, but also shows that it appears to be dispensable for the response against cell wall perturbing agents, an unusual finding in fungi which suggests uncoupling between these stress responses in *N*. *crassa*. In addition, we found that growth on cellulose, the most abundant component of plant biomass and a natural substrate of Neurospora in the wild, imposes high demands on ER function and is dramatically impaired in the absence of HAC-1, pointing towards a physiological role for the UPR in this fungus. Our data thus support a specific role for HAC-1 in the UPR in *N*. *crassa* and for this transcription factor in cellulose catabolism, highlighting a basic cell signaling pathway that can be further manipulated-considering the variety of molecular tools available in *N*. *crassa*- to provide new insights into the biotechnological applications of this filamentous fungus, particularly in the context of the bioconversion of lignocellulosic material to simple sugars for biofuel production.

## Results

### Identification and characterization of *N*. *crassa hac-1*

**Fig 1:**
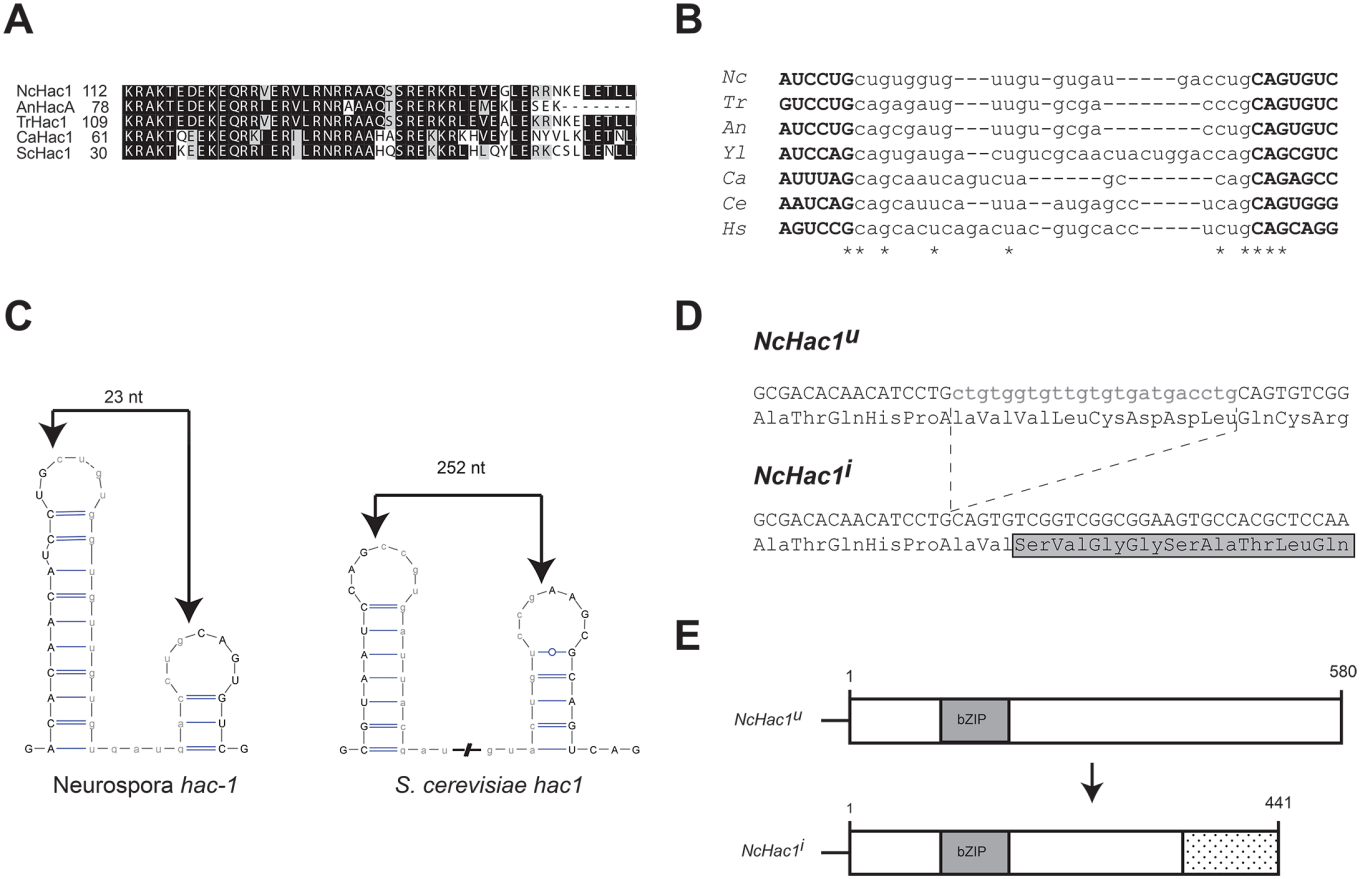
*Neurospora crassa* has a putative Hac1 homolog. A) Alignment of the amino acid sequence of the bZIP domain of selected fungal HAC-1 homologs. *Nc*: *Neurospora crassa*; *An*: *Aspergillus nidulans*; *Tr*: *Trichoderma reseei*; *Ca*: *Candida albicans; Sc*: *Saccharomyces cerevisiae*. B) Sequence alignment of the putative non-conventional intron and surrounding regions of *hac-1* homologs in fungi. Asterisks denote positions conserved in all the sequences considered in the alignment. *Nc*: *Neurospora crassa*; *Tr*: *Trichoderma reseei*; *An*: *Aspergillus nidulans*; *Yl*: *Yarrowia lipolytica*; *Ca*: *Candida albicans*; *Ce*: *Caenorhabditis elegans*; *Hs*: *Homo sapiens*. C) Predicted twin stem-loop structure of the intron of the *N*. *crassa* and yeast *hac-1* mRNAs. Folding prediction was made with mFOLD [76] and the structures drawn with VARNA [77]. Predicted cleavage sites are indicated by an arrow. The predicted intron sequence is shown in lowercase. D) Nucleotide and deduced amino acid sequence surrounding the putative splice sites in the *N*. *crassa hac-1* CDS. Upon ER stress, the putative intron (shown in lowercase) is removed from the uninduced version of the *hac-1* mRNA (*hac-1* ^*u*^), resulting in the induced version (*hac-1* ^*i*^). Translation of the induced version would alter the reading frame, leading to a different C-terminal region. E) Schematic representation of NcHAC-1^u^ and NcHAC-1^i^.

A BLASTP search of the *N*. *crassa* genome, using the *S*. *cerevisiae* Hac1 protein sequence as query, revealed *NCU01856* as a putative ortholog of this gene. The predicted *N*. *crassa* protein, herein referred to as HAC-1, is composed of 580 amino acids and contains a conserved bZIP domain, highly similar to the one present in other characterized Hac1p homologs from different fungal species (Fig 1A). In addition, a small element in the 3’ UTR of the *S*. *cerevisiae hac1* mRNA, which has been shown to be important of its splicing *in vivo* [39], also appears to be conserved in the identified *N*. *crassa hac-1* gene.

The *S*. *cerevisiae HAC1* mRNA undergoes a non-spliceosomal splicing reaction under ER stress, catalyzed by the kinase/RNAse Ire1p and the tRNA ligase Trl1/Rlg1p, which results in the removal of a 252 nt intron. In filamentous fungi and interestingly also in the yeast *Candida albicans*, shorter introns (~20 nt long) have been reported for the *Hac1* homologs. By comparing the sequence of *hac-1* with those in different fungal species, we predicted that under ER stress, an intron of 23 nt would be removed from the *hac-1* mRNA. This is based on the known consensus splice sites for the unconventional intron in various organisms (Fig 1B). While in other filamentous fungi, the exact position of the intron could not be unambiguously determined due to the presence of a CTGCAG segment at each side of the intron [22, 23, 28], the predicted *N*. *crassa* intron sequence is asymmetrically flanked, with CTGCTG on the 5’ region and CTGCAG on the 3’ end (Fig 1B), bordering a 23 nt intron. Further supporting a 23 nt unconventional intron in the *N*. *crassa hac-1* mRNA, is its predicted secondary structure and surrounding sequences. IRE-1 targets exhibit a similar predicted RNA secondary structure, consisting of twin hairpins in which the cleavage sites reside on the loops [40]. The lowest free energy form of *hac-1* mRNA also conforms to this structure, similar to its counterparts in different organisms, with the predicted cleavage sites located on the loops and surrounding the predicted 23 nt intron (Fig 1C). Removal of this intron in the *N*. *crassa hac-1* sequence would alter the reading frame (Fig 1D) leading to the production of a predicted shorter protein (Fig 1E). Moreover, and consistent with a phylogenetically conserved mechanism, the *N*. *crassa* genome harbors, in addition to *hac-1*, genes encoding for the putative homologs of Ire1p, Rlg1p and Ptc2 (S2 Table).

**Fig 2:**
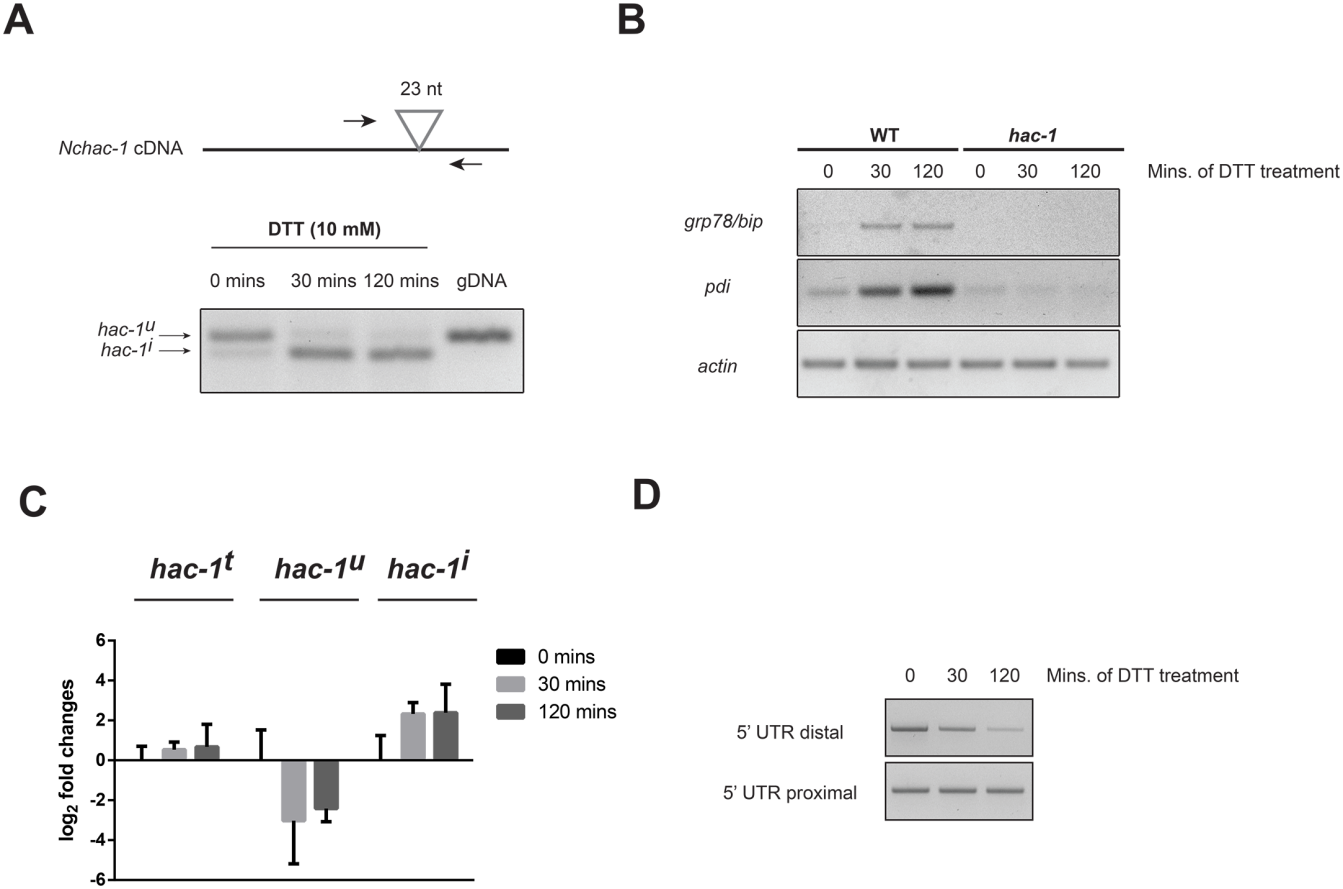
The *N*. *crassa hac-1* mRNA is processed under ER stress. A) (Top) Schematic representation of the position of the primers used to detect the processing of the unconventional intron in the *N*. *crassa hac-1* mRNA. The position of the intron is shown as a triangle. (Bottom) RT-PCR analysis from WT strain (FGSC #988) to detect processing of the *N*. *crassa hac-1* mRNA upon chemically-induced ER stress. Amplification using the same primers, but with genomic DNA (gDNA) as template, is shown for size comparison. B) RT-PCR analysis to detect expression of homologs of typical UPR target genes, *grp78*/*bip* (*NCU03982*) and *pdi* (*NCU09223*), under ER stress in both WT and Δ*hac-1* strains. Amplification with primers for *actin* mRNA was used as a loading control. C) Real-time quantitative PCR analysis of the expression levels of the distinct isoforms of *hac-1* mRNA (t: total; u: uninduced; i: induced) under ER stress with DTT for the times depicted. Bars represent mean expression values +/- 95% confidence intervals, from 3 independent biological replicates. D) RT-PCR analysis for detecting changes in the length of the 5’ UTR of the *N*. *crassa hac-1* transcript upon ER stress. Primers targeting a 5’ UTR region close to the start codon (5’ UTR proximal) and a region further upstream (5’ UTR distal) were used. Assays were performed on 3 biological replicates per condition with similar results.

To determine whether the *N*. *crassa hac-1* mRNA is indeed processed in response to ER stressing agents, *N*. *crassa* liquid cultures were treated with the reducing agent dithiothreitol (DTT) for 30 and 120 minutes. DTT is known to induce ER stress by altering the oxidative environment in the ER and disrupting disulfide bonds, thus leading to the accumulation of misfolded proteins. By designing primers flanking the predicted splice site (Fig 2A, top), it is therefore possible to assess whether the mRNA is processed and the predicted unconventional intron removed, in an ER stress-dependent manner in *N*. *crassa*. Indeed, as shown in Fig 2A (bottom), the *hac-1* mRNA is rapidly and efficiently processed in response to DTT, whereas almost no processing is observed in untreated controls. The conditions used to detect this processing of the *hac-1* mRNA indeed elicit the unfolded protein response in *N*. *crassa*, as evidenced by the induction of predicted UPR targets *grp78/bip* and *pdi*, predicted homologs of genes encoding an ER-resident class HSP70 chaperone and protein disulfide isomerase, respectively, which are known to be up-regulated in different species under ER stress (Fig 2B) [41]. Sequence comparison between *hac-1* cDNA derived from untreated and DTT-treated cultures, confirmed that the observed processing (Fig 2A) corresponded exactly to the removal of the predicted unconventional intron of 23 nt (Fig 1B).

Consistent with the induction of ER-stress genes, we also observed that in response to DTT, there was a rapid increase in the levels of the spliced form of *hac-1* mRNA, as the levels of the unspliced one fell (Fig 2C). Total *hac1* mRNA levels on the other hand, remained relatively unchanged under our conditions of chemical ER stress, suggesting that the acute response to misfolded proteins via the HAC-1 pathway operates mainly through post-transcriptional regulation of the *hac-1* mRNA in *N*. *crassa*.

A truncation of the 5’ end of the *hac-1* transcript has been reported to take place upon ER stress in different fungal species with short unconventional introns [22, 23, 42]. To evaluate whether such regulation exists in *N*. *crassa*, we designed an RT-PCR assay to detect ER stress-induced changes in the length of the 5’ UTR region of the *hac-1* mRNA. By designing forward primers that anneal to one of two locations within the 5’ UTR (one very close to the start codon and one further upstream) and a common reverse primer within the *hac-1* coding region (S1 Table), we show that the abundance of the *hac-1* mRNA population that harbors a long 5’ UTR is reduced upon ER stress, while the amount of total *hac-1* transcript, as assessed with the reaction targeting the proximal 5’ region, is unaffected (Fig 2D), suggesting that a truncation in the 5’ UTR region of the *N*. *crassa hac-1* transcript takes place in the presence of ER stress in a region that is upstream of position -66.

### Functional evaluation of *hac-1*

In order to evaluate the role of *hac-1* in coping with ER stress and as possible regulator of the unfolded protein response in *N*. *crassa*, we proceeded to characterize a *hac-1* knockout (KO) strain. Since the KO was not available from the *N*. *crassa* KO collection [36], we generated one by replacing the *hac-1* coding region with a drug resistance cassette. Correct integration of the cassette and replacement of the *hac-1* sequence was verified by PCR as shown in S1 Fig.

**Fig 3:**
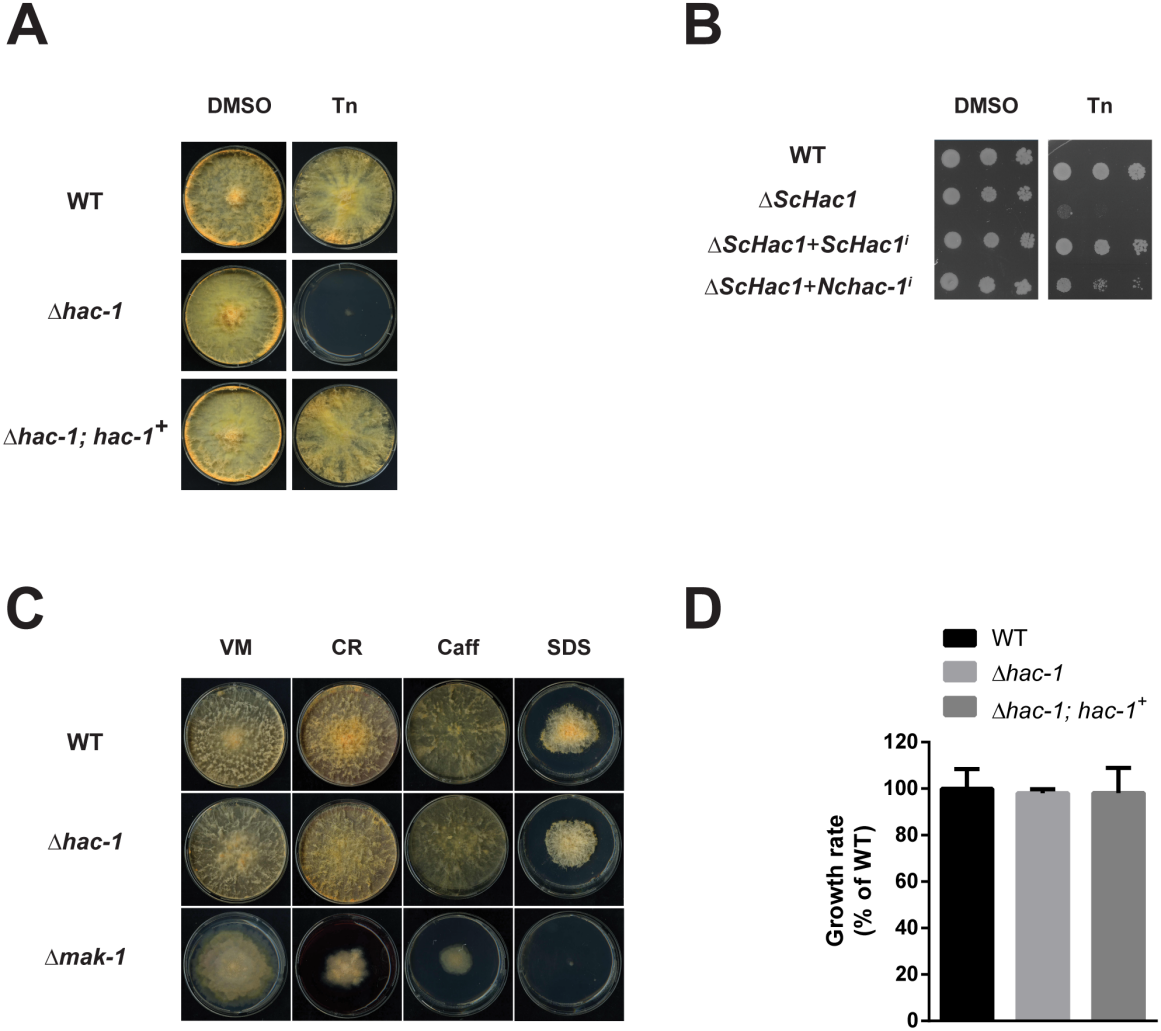
The *hac-1* gene is necessary to cope with ER stress in *N*. *crassa*, but is dispensable for cell wall stress. Conidia from WT (FGSC #988), Δ*hac-1* and **Δ** *hac-1* complemented with a WT copy of *hac-1* (Δ*hac-1*; *hac-1* ^+^), were inoculated on solid Vogel’s media (VM) with or without Tunicamycin (Tn). Plates were placed for 3 days at 25°C in constant light. DMSO was used as a vehicle control for Tn. B) Viability assay by serial dilutions of *S*. *cerevisiae* Δ*hac1* strains expressing either the yeast or the *N*. *crassa hac-1* induced versions under the control of the *S*. *cerevisiae ADH1* promoter. Ten-fold serial dilutions of logarithmic-phase cells were spotted onto SC-leu agar plates in the presence and absence of 0.2 μg/ml Tn and the plates were incubated for 6 days at 30°C. C) Conidia from WT (FGSC #988), Δ*hac-1* and Δ*mak-1* (FGSC #11321) strains were inoculated on solid Vogel’s media with or without the chemicals shown. CR: congo red; Caff: caffeine; SDS: Sodium Dodecyl Sulfate. Plates were placed for 3 days at 34°C in constant dark and then grown for an additional 24 h in constant light at 25°C before imaging. All phenotypic assays were performed at least 3 independent times with similar results. D) Conidia from the strains described in the figure were inoculated on race tubes containing solid Vogel’s media and were then placed under constant light conditions at 25°C for 5 days. Marks were done on the tubes every 24 h and the distance between the marks was used to calculate the linear growth rate per day. Bars represent mean expression values +/- 95% confidence intervals, from 4 independent biological replicates.

Growth of the *hac-1* mutant was relatively normal under non-stressful conditions (Fig 3A), although it exhibited a slightly lower growth rate during the first day after inoculation on race tubes (a difference that was, however then lost on subsequent days, Fig 3D). We then evaluated growth of this strain under chemically-induced ER stress and as shown in Fig 3A, disruptants of *hac-1* displayed enhanced sensitivity to Tunicamycin (an inhibitor of N-linked glycosylation that leads to ER stress), compared to the WT. Such sensitivity to ER stress displayed by the mutant was reverted by the reintegration of a WT copy of the *hac-1* gene at the endogenous locus, thereby confirming that the HAC-1 transcription factor is required for coping with ER stress in *N*. *crassa*.

As mentioned previously, up-regulation of *grp78/bip* and *pdi* are part of the typical expression signature of the unfolded protein response in several organisms. To test whether the elevated mRNA levels observed for these genes under ER stress conditions in *N*. *crassa* (Fig 2A) depend on HAC-1, we evaluated their expression in the Δ*hac-1* strain. As shown in Fig 2B, while the transcript levels of *grp78/bip* and *pdi* rapidly rise in response to ER stress in the WT, no induction is observed in the Δ*hac-1* strain, suggesting that this transcription factor is important for their up-regulation under ER stress in *N*. *crassa*. Consistent with this, analysis of the promoter region of both of these genes reveals matches to the known *cis*-acting unfolded protein response element cUPRE-1 (S2 Fig) [43].

The *N*. *crassa hac-1* gene thus displays typical characteristics of a *HAC1* homolog: it is required for growth under ER stress and for the induction of classic ER stress-responsive genes. We furthered our functional characterization of *hac-1* by performing yeast complementation assays, to evaluate whether the *N*. *crassa* gene can functionally replace the yeast *HAC1* gene. To accomplish this, we cloned the induced form of *HAC1/hac-1* cDNA from both *S*. *cerevisiae* and *N*. *crassa* and placed them under control of the constitutive yeast *ADH1* promoter. Constructs were then transformed into the *S*. *cerevisiae ΔHAC1* strain and cells were spotted on solid media with or without Tunicamycin. As expected, the yeast *ΔHAC1* strain displayed a growth defect when grown on media supplemented with this drug, a phenotype that is reverted by the introduction of the induced form of the WT yeast *HAC1* gene (Fig 3B). Likewise, when the induced form of the *N*. *crassa hac-1* gene was expressed in this mutant, the yeast *ΔHAC1* strain recovered its ability to grow in the presence of this ER stressing agent, showing that the *N*. *crassa hac-1* gene can functionally replace its yeast counterpart.

A number of studies have revealed a link between UPR function and cell wall homeostasis in fungi, such that *hac1* or *ire1* deletion strains usually display enhanced sensitivity to cell wall perturbing agents (reviewed in [17]). As part of our characterization of *hac-1*, we thus decided to evaluate growth of the *N*. *crassa* Δ*hac-1* strain under these conditions, using the Δ*mak-1* strain as a control for cell wall stress sensitivity, as the *mak-1* gene, which encodes for a MAPK that is homologous to the yeast Slt2 MAPK, has been shown to be involved in the regulation of cell wall integrity in *N*. *crassa* [44]. As shown in Fig 3C, we observed no increased sensitivity of the mutant to the glycan-binding agent Congo Red, caffeine or the ionic detergent SDS, all of which have been used extensively to test for cell wall sensitivity in fungi [45, 46], suggesting that the connection between the UPR and cell wall integrity pathways described in some fungi, may be different in *N*. *crassa* or that other mechanisms can compensate for the absence of *hac-1* for maintaining cell wall homeostasis in this fungus.

Taken together, our results indicate that *N*. *crassa hac-1* represents a functional homolog of the yeast *HAC1* gene and that it plays an important role in coping with ER stress and in mediating transcriptional responses under these conditions in *N*. *crassa*. In addition, these results highlight differences in the role of a widely conserved transcription factor in different fungal species.

### The *N*. *crassa hac-1* gene is required for growth on cellulose

We surmised that *N*. *crassa* growth under complex carbon sources, a process requiring extensive adaptation of the secretion machinery for the production of numerous enzymes, could lead to ER stress and hence, would require HAC-1. To test this idea in *N*. *crassa*, we evaluated growth of WT and Δ*hac-1* mutant strains on crystalline cellulose (Avicel) and hemicellulose (xylan), as degradation of these types of substrates requires the secretion of a large variety and quantity of enzymes [35, 47–50]. Interestingly, this would provide an attractive “natural” test for ER stress, considering that in its natural environment, *N*. *crassa* degrades plant biomass and has in fact emerged as a premier model system for studying plant cell wall deconstruction by filamentous fungi in the last few years [35, 37, 49, 50].

**Fig 4:**
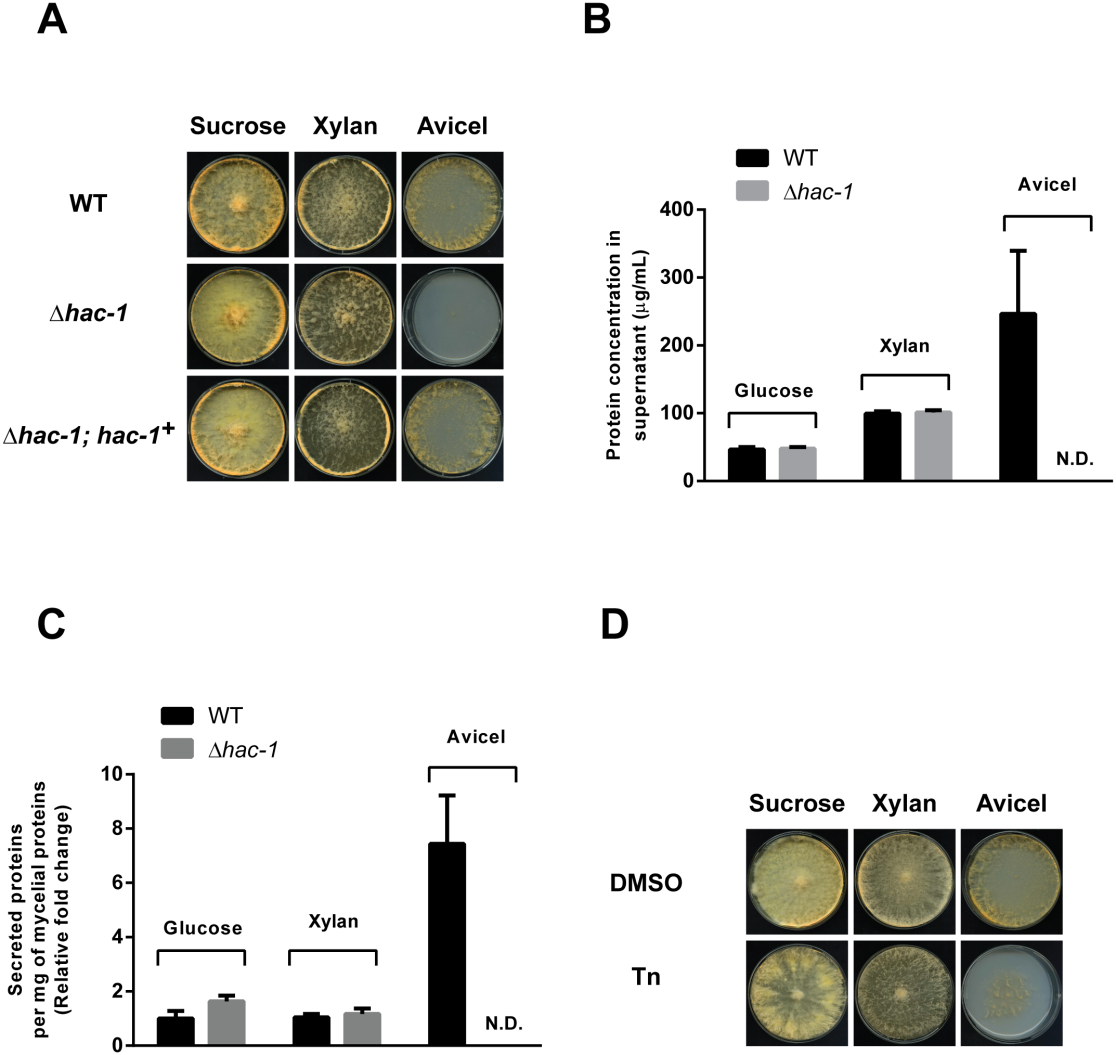
HAC-1 is required for growth on cellulose. **A)** Conidia from WT (FGSC #988), Δ*hac-1* and Δ*hac-1* complemented with a WT copy of *hac-1* (Δ*hac-1*; *hac-1* ^+^), were inoculated on solid Vogel’s media with sucrose, xylan or crystalline cellulose (Avicel) as carbon source (2% w/v). Plates were placed for 4 days at 25°C in constant light before imaging, except the Avicel plates, which were photographed after 6 days under the same conditions. B) Protein concentration from supernatants of cultures of WT and Δ*hac-1* strain growing on different carbon sources for 7 days, were determined. Bars represent mean expression values (+/- S.E.) from 3 independent biological replicates. N.D. not detected. C) The protein concentration from supernatants was normalized to the total amount of mycelial proteins per condition, and is expressed relative to the ratio exhibited by the WT growing on glucose. Bars represent mean expression values (+/- S.E.) from 3 independent biological replicates. D) Conidia from WT (FGSC #988) were inoculated on solid Vogel’s media with sucrose, xylan or crystalline cellulose (Avicel) as carbon source (2% w/v), with or without Tunicamycin (Tn). Plates were placed for 3 days at 25°C in constant light before imaging, except the Avicel plates, which were photographed after 4 days under the same conditions. All phenotypic assays were performed at least 3 independent times with similar results.

As shown in Fig 4A, growth on solid media with Avicel as the sole carbon source is dramatically impaired in the absence of *hac-1*, while growth on xylan appears to be normal compared to sucrose, suggesting that a particular aspect of growth, specific to growth on cellulose, requires HAC-1. Similar results were obtained under liquid culture conditions (S3 Fig). This growth phenotype exhibited by Δ*hac-1* is suppressed by reintroducing a WT copy of *hac-1* (Fig 4A, lower panel). We reasoned that growth on cellulose may be impaired in the *hac-1* mutant strain, compared to xylan (and sucrose), due to an increase in protein secretion and/or folding requirements on this carbon source over the others. Indeed, quantification of protein concentration from supernatants of the WT strain growing on Avicel, glucose or xylan, revealed that the amount of protein secreted on Avicel is higher that on the other two carbon sources (Fig 4B). This is also observed after accounting for the amount of biomass present under each condition (Fig 4C). In this scenario, growth on Avicel could then impose higher demands on ER function than growth on the other two carbon sources. Consistent with this idea, WT strains growing on Avicel display enhanced sensitivity to chemically-induced ER stress (Fig 4D), suggesting that simply growing on cellulose constitutes a basal stressful condition for the ER in *N*. *crassa*. Coping with such carbon source-related stress, a condition likely encountered by *N*. *crassa* in the wild, would require HAC-1. Quantification of the protein concentration from supernatants of the *hac-1* strain growing on Avicel, glucose or xylan, shows that while no protein is detected on Avicel (which basically reflects its severe growth phenotype), the amount of protein secreted on glucose and xylan appears to be normal compared to that of the WT (Fig 4B and 4C), suggesting that a global secretion problem is unlikely to be the main cause of the growth defect of this strain on Avicel and again suggests a specific defect associated with cellulose catabolism.

Taken together, our results indicate that the *N*. *crassa hac-1* gene and hence a functional UPR, is required for growth on cellulose, highlighting a natural scenario in which the unfolded protein response is required in *N*. *crassa* in the wild.

## Discussion

The unfolded protein response (UPR) signaling pathway has been intensively studied on a variety of systems since its first report over 20 years ago in *S*. *cerevisiae* [11, 12, 51]. In mammalian models, alterations in the UPR have been associated with several pathologies and syndromes [52], while UPR components have been also highlighted as a potential target for anticancer therapies [53, 54]. From a biotechnological point of view, the fungal UPR has attracted attention mainly with the goal of overcoming bottlenecks associated with the industrial production of heterologous proteins, as well as with the production of endogenous proteins of interest, particularly using *T*. *reesei* and different Aspergillus species [19–21]. In addition, in the past few years, the fungal UPR has been shown to impact virulence of several human and plant pathogens [31, 34, 55].

Despite the plethora of molecular tools available in *N*. *crassa* and its long-tradition as a model for several cell biology processes, the UPR has not been studied in this organism. Moreover, in previous compendiums of putative transcription factors encoded in the *N*. *crassa* genome [38, 56], the gene model encoding for HAC-1 (*NCU01856*) was not listed neither as a transcription factor nor as a putative *hac-1* homolog. Therefore, we set out to characterize the UPR in *N*. *crassa*, particularly focusing on HAC-1 and its role in this signaling pathway.

We identified and characterized the ortholog of the yeast *HAC1* gene in the filamentous fungus *N*. *crassa*, *hac-1*. We show that the *N*. *crassa hac-1* gene can complement the growth defect exhibited by a yeast *HAC1* mutant under ER stress. While complementation is not complete, such partial reversion of the mutant phenotype after introduction of a *Hac1* ortholog is common [32, 33, 42] and may be due to a variety of reasons (e.g. codon usage, folding requirements, missing interacting partners, etc.). During the course of this work, we further demonstrated that *NCU01856* is a sequence-specific DNA binding protein [57] and that its motif resembles the one determined for the yeast Hac1 protein, consistent with their similarity in the DNA binding domain [57, 58]. Sequence preference information is available at the Cis-BP database (http://cisbp.ccbr.utoronto.ca/).

As expected for a *HAC1* ortholog, the *N*. *crassa hac-1* mRNA has a well conserved consensus structure and it undergoes an ER stress-dependent splicing reaction, which removes a 23 nt intron. The size of this intron has recently been confirmed by another group [59]. Such intron sizes in *HAC1* homologs (~20 nt) are typical among filamentous fungi and metazoans. Interestingly, most studied yeast species appear to have long ones (> 100 bases), with few exceptions [40]. In *S*. *cerevisiae*, a long intron is involved in translational attenuation of the *HAC1* mRNA in the absence of ER stress via base-pairing between the intron and the 5’UTR [60, 61]. In fungal species which lack such long intron and have a short one instead, unable of such extended base-pairing, different mechanisms regulating *HAC1* expression and activity have been suggested to be at play, including translational regulation via modulation of the length of the 5’ UTR. Such mechanism may similarly play a role in *N*. *crassa*, in which the length of the 5’ end of the *hac-1* transcript appears to be modulated in response to ER stress, as it does in *C*. *albicans*, *A*. *nidulans*, *A*. *niger* and *T*. *reesei* [22, 23, 42]. The mechanisms regulating this change in the length of the 5’UTR (e.g. alternative transcription initiation site or post-transcriptional processing of the *hac-1* transcript upon ER stress) are unknown.

By disrupting *hac-1*, we were able to assess its contribution to the UPR and growth under ER stress conditions in *N*. *crassa*. We found that *N*. *crassa* HAC-1 is necessary for the up-regulation of typical UPR targets when cultures are subjected to ER stress and that HAC-1 is necessary for growth under such conditions. While HAC-1 orthologs are usually shown to be required for growth under ER stress conditions, the specific phenotype resulting from the deletion of HAC1 homologs, in the absence of such stress, depends on the organism under study. For example, in *A*. *niger*, loss of *hacA* leads to growth and developmental defects on rich media, in the absence of ER-stress inducing agents [62]. Similarly, loss of *HAC1* has been shown to impact morphology in *C*. *albicans* [42]. Disruptants of *hacA* in the filamentous fungus *A*. *fumigatus* and *Alternaria brassicicola* on the other hand, show normal radial growth on rich media in the absence of ER-stressing agents [28, 32], highlighting that no *a priori* conclusions can be reached regarding the phenotype of a loss-of-function *hac-1* mutant in fungi, even among closely related species. Here we show that the *N*. *crassa hac-1* mutant exhibits relatively normal growth on media containing simple sugars as carbon source, both on liquid and solid settings, in the absence of ER-stressing agents. Notably however, in the course of characterizing the *N*. *crassa* HAC-1 transcription factor in the context of the UPR, we found that growth of the *N*. *crassa hac-1* mutant is severely impaired on crystalline cellulose, suggesting a paramount role for HAC-1 (and possibly the UPR) in the deconstruction of this substrate found in the wild. Our study thus broadens the range of physiological functions of the UPR in fungi. Interestingly, growth on xylan, which also constitutes a rather complex carbon source, is unaffected in the mutant, suggesting a specific defect on cellulose metabolism. Notably, such a particular growth defect resembles the one described for strains lacking the cellulose-specific transcription factors CLR-1 and CLR-2 [63]: similar to Δ*hac-1*, these mutants exhibit growth defects on Avicel, but not on xylan, suggesting that a particular aspect of growth, specific to growth on cellulose, requires HAC-1. Our findings highlight a basic, conserved and well-known signaling pathway whose manipulation can have important biotechnological implications on the industrial use of *N*. *crassa* for protein production and biofuels research, the latter of which relies on the efficient bioconversion of lignocellulosic biomass to simple sugars.

The preliminary finding herein reported of the growth defect exhibited by the *hac-1* mutant on cellulose, raises the question as to why this strain is unable to deconstruct and grow on cellulose, opening an interesting area of research on its mechanistic basis, considering the growing use of *N*. *crassa* in the study of cellulose deconstruction [35]. A number of ideas can be proposed, none of which are mutually exclusive. The most intuitive explanation, considering the role of HAC-1 in the unfolded protein response, concerns a compromised secretory pathway capacity resulting from the inability of the mutant strain to mount the UPR under particular high ER function-demanding conditions. In mammals, several secretory cell types have been shown to rely on a functional UPR for differentiation and function [64]. Further, while deletion of *hacA* in *A*. *fumigatus* displays normal growth on rich media, this gene has been shown to be required for proper growth on a relatively complex substrate such as skim milk [28] and a similar result was also reported for the corresponding mutant in *A*. *brassicicola* [32]. Indeed, our results suggest that growth on Avicel per se imposes ER stress on *N*. *crassa*. It has been reported that *N*. *crassa* secretes large amounts of a variety of enzymes upon transfer to Avicel as a carbon source (compared to sucrose) [50] and higher secretion demands on this carbon source, with a concomitant requirement for a fully active and functional UPR, may partly explain the obvious growth defect exhibited by the *hac-1* mutant growing on Avicel compared to the other two tested carbon sources. The data herein presented attest to this comparatively increased protein secretion on Avicel. Interestingly, a recent study [59] has shown that several genes likely involved in the ER stress response and the secretory pathway in *N*. *crassa*, are specifically induced by growth on Avicel (and not on other carbon sources like xylan), which is consistent with our results. Further, among the genes within the *N*. *crassa* Avicel regulon, that is, genes that specifically respond to this carbon source [63], are well characterized proteins with secretory functions, again suggesting that cellulose metabolism requires significant accommodation of the secretory pathway as a whole in this fungus. In addition, and consistent with our interpretation, it has been reported both in Trichoderma and only recently in *N*. *crassa* (while this work was in preparation), that induction of cellulase production leads to UPR activation [59, 65]. Interestingly, up-regulation of *hacA* has been observed in *A*. *nidulans* during growth on lignocellulose [66].

Higher demands for proper ER function on Avicel may not only be related to an increased protein flux through the ER, but they could also derive from the fact that some of the specific enzymes involved in the metabolism of this carbon source may have high folding and/or processing/modification requirements, thus demanding a functional UPR to accommodate them in large quantities [67]. It could also be proposed that growth under Avicel is impaired in the *hac-1* strain due to an overall secretory pathway problem. For instance, deletion of *vib-1*, a gene required for extracellular protease secretion in response to carbon and nitrogen starvation in *N*. *crassa* [68], results in growth defect and reduced extracellular enzyme activity, on both cellulose and xylan [69]. In addition, loss of *hacA* in *A*. *brassicicola* results in an overall reduced secretion capacity [32]. Our data however, suggest that a global secretory pathway defect may not be the main cause of the growth phenotype of the Δ*hac-1* strain, as the amount of proteins secreted by this mutant appears to be normal on both glucose and xylan, the latter, a carbon source that induces the secretion of a variety of enzymes [49]. This is consistent with our phenotypic data, as growth on these media is unaltered in the mutant and further highlights differences in the functions of HAC-1 among fungal species. Alternatively, a general secretory problem might indeed be present, but it would only be observable when a particular threshold in ER capacity/secretory load is surpassed (integrating increased protein flux, folding requirements, etc.) and in our conditions, such a threshold would only be met under Avicel and not xylan or glucose. In any case, under non-stressing conditions, the hyphal growth rate between WT and the *hac-1* strains are similar, even though hyphal growth is accompanied by the trafficking of numerous vesicles to the hyphal tip. This is noteworthy, as deletion of *HAC1* has been shown to affect polarized growth in *C*. *albicans*, even in the absence of ER stress, which has been suggested to result from alteration of vesicular trafficking [42]. In addition, while an efficient secretion system is also important for cell wall biosynthesis [17], we observed that the Δ*hac-1* strain was not sensitive to cell wall perturbing agents, again suggesting that a general secretion problem is an unlikely explanation for the bulk of the growth defect exhibited by the *hac-1* strain on Avicel. This is an unusual finding in fungi, as inactivation of this bZIP transcription factor has been shown to affect cell wall integrity in various fungal species [17, 28, 32, 42]. The observation then, that the *N*. *crassa* Δ*hac-1* strain is capable of normal growth under chemical cell wall stress, merits further investigation, as it highlights differences in the cellular roles of *hac-1* in different species. Finally, it is also possible that *hac-1* specifically regulates the expression of enzymes involved in the deconstruction of cellulose, which could explain the phenotype observed in its absence on that carbon source, an idea currently under evaluation by various laboratories, prompted by results herein presented.

We have done an initial characterization of the unfolded protein response in *N*. *crassa*, focusing on the bZIP transcription factor HAC-1. Despite decades as a research model system, such conserved regulatory pathway had not hitherto been studied in this fungus and we herein show it to display the core conserved aspects of the response. Despite this conservation and the fact that HAC-1 and the UPR have been studied in various fungal species, we report on aspects that at present, seem to be uncommon among fungi: the *N*. *crassa hac-1* gene appears to be dispensable for growth under cell wall stress conditions and its ablation does not appear to dramatically affect global secretion. In addition, we found that HAC-1 is necessary for growth on cellulose, a substrate encountered by Neurospora in the wild, a finding that expands the range of physiological functions of the UPR known in fungi. Further studies on this system and its manipulation, considering all the molecular and genetic tools available in this model fungus, will help not only in the study of the unfolded protein response, which can now be studied on a natural setting (rather than relying on chemical insults), but will also provide new insights into the basic biology behind plant biomass deconstruction by fungi, a field that is rapidly growing [35, 37] and that has an undeniable impact in both applied and environmental biological research. Additionally, these studies, will provide not only a deeper understanding of the different mechanisms regulating protein synthesis and secretion in this organism, but also of fungal growth on a commercially relevant substrate such as cellulose, shedding light on research focused on the improvement of industrial cellulase production for biofuel generation using *N*. *crassa* [37], an area which would benefit from a better understanding of the molecular mechanisms regulating the expression of hydrolytic enzymes.

## Methods

### Strains

The general conditions for growth and maintenance have been previously described [70]. Wild-type strain FGSC#988 (Mat a) was used for all phenotypic and gene expression assays. A *hac-1* knockout strain (Mat a) was generated by disrupting the *NCU01856* ORF through targeted gene replacement [71] with a bacterial bialaphos-resistance (*bar*) gene (which confers resistance to Ignite), followed by homokaryonization via sexual crossing. Correct integration of the cassette was verified by PCR (S1 Fig and S1 Table). The Δ*hac-1; hac-1* ^+^ complemented strain was generated by transforming the aforementioned *hac-1* knockout strain with a cassette, targeted at the endogenous locus (so that it would replace the KO cassette by homologous recombination) and conferring resistance to hygromycin, containing the full *hac-1* gene sequence, followed by the *actin* gene (*NCU04173*) transcriptional termination sequence. Primer sequences are listed in S1 Table.

### Culture conditions for gene expression assays

Conidia from WT and *hac-1* KO strains (10^5^ conidia/mL) were inoculated into flasks containing liquid Vogel’s medium (pH 5.8) with 2% (wt/vol) glucose, 0.5% arginine and 50 ng/mL biotin. Flasks were kept in incubators (Percival Scientific) in constant light at 25°C and were shaken at 125 rpm. After 2 days, a 1M stock solution of dithiothreitol (DTT) was added directly to each culture, to a final concentration of 10 mM for the times described in the Figure legends. Mycelia was harvested, dried and then wrapped in aluminium foil. Mycelia was then rapidly frozen in liquid nitrogen and stored at -80°C.

### RNA extraction and RT-PCR

Total RNA was prepared essentially as described by Kramer [72]. The concentration of each RNA sample was measured using the Nanodrop 2000 Spectrophotometer (Thermo Scientific). All of the RNA samples had a 260/280 ratio and a 260/230 ratio of ≥2. RNA integrity was verified on 1% agarose gel with ethidium bromide staining. Prior to cDNA synthesis, RNA samples were treated with RQ1 RNase-free DNase (Promega) according to manufacturer’s instructions. For end-point RT-PCR, 0.25 μg of DNAse-treated RNA was reverse-transcribed using M-MLV (Promega) and oligo-dT, according to manufacturer’s instructions. For RT-quantitative PCR, reverse transcription was performed on 0.5 μg of DNAse-treated RNA using SuperScript III (Invitrogen) and anchored oligo-dT, according to manufacturer’s instructions. The reaction was subsequently diluted 10 times with nuclease-free water (Life Technologies) and used for real-time quantitative PCR (qPCR).

### Real-time PCR

Real-time quantitative PCR was performed in the StepOnePlus system (Applied Biosystems) in a 96-well plate format. Each qPCR reaction contained the SensiMix SYBR Hi-ROX mix (Bioline Inc. USA) (6.25 μL), 0.25 μL of a mix of specific forward and reverse primers (10 μM each), 1 μL nuclease-free water and 5 μL of cDNA (2.5 ng/μL of RNA equivalents). The cycling conditions were as follows: 10 min at 95°C and 40 cycles of 15 s at 95°C, 15 s at a 66°C (which was determined as an optimal annealing temperature for all the primer pairs used) and 15 s at 72°C, followed by melt curve analysis (ran from 60°C to 95°C with 0.3°C increments). Primer specificity was evaluated by both melt curve and agarose gel analyses. Primer sequences for the quantification of the different *hac-1* isoforms are listed in S1 Table. *Actin* was used as a reference gene for normalization. All reaction efficiencies were between 90–100%. Three independent biological replicates per conditions were used.

### Phenotypic assays

For chemically-induced ER stress assays, conidia (10^6^) from WT (FGSC#988), Δ*hac-1* and Δ*hac-1; hac-1* ^+^, were inoculated on plates containing solid Vogel’s media (1X Vogel’s salts, 2% sucrose, 1.5% agar), supplemented with 0.4 μg/mL Tunicamycin or vehicle (DMSO). Plates were grown in constant light at 25°C for the number of days described in the corresponding Figure legends before imaging. For growth assays under different carbon sources, conidia were inoculated on solid media containing 1X Vogel’s salts, 0.5% arginine, 50 ng/uL biotin and 1.5% agar, supplemented with either 2% sucrose, 2% Avicel PH-101 (Sigma-Aldrich 11365) or 2% Xylan from beechwood (Sigma-Aldrich X4252). To test for hypersensitivity to chemical ER-stressing agents under these carbon sources, the aforementioned media was supplemented with 0.4 μg/mL Tunicamycin or vehicle (DMSO). Plates were incubated in constant light at 25°C for the number of days mentioned in the corresponding Figure legends before imaging. Imaging of Avicel plates was always performed later than for the other media, as strains grow relatively slower on this carbon source. For cell wall sensitivity assays, conidia from WT (FGSC #988), Δ*hac-1* and Δ*mak-1* (FGSC #11321) were inoculated on solid Vogel’s media supplemented with Congo Red (200 μg/mL), caffeine (5 mM) and SDS (0.01% w/v), which are known fungal cell wall stress inducers [45, 46]. All phenotypic assays were repeated at least 3 independent times.

### Yeast complementation assay

The full coding sequence of the yeast *hac1* gene and the *N*. *crassa hac-1* gene was amplified from cDNA obtained under ER stress conditions and cloned by yeast recombinational cloning [73], together with the in-frame sequence for a C-terminal V5 tag, into the integrative plasmid pGAD424 (Clonetech), after its linearization with *Kpn*I and *Bgl*II,. This placed the cDNA sequences under control of the yeast *ADH1* promoter. Yeast were transformed using the LiAc/SS carrier DNA/PEG method [74] and selected on SC-leu media. For the phenotypic assay, logarithmic-phase cells were adjusted to OD_600_ 0.4 and 2 μl of serial 10-fold dilutions were spotted onto SC-leu agar plates in the presence and absence of 0.2 μg/ml Tunicamycin and the plates were incubated for 6 days at 30°C. Assays were repeated 3 independent times.

### Protein quantification

Conidia from WT (FGSC#988) and Δ*hac-1* strains were inoculated into flasks containing liquid Vogel’s medium (pH 5.8) and grown as described above, with 2% (wt/vol) of the carbon source of interest (glucose, xylan or Avicel) for 7 days. After that time, the amount of secreted proteins per condition was measured using the Bradford assay (BioRad), using 100 μL of culture supernatant. The amount of mycelial proteins was determined as in [75]. The assay was performed 3 independent times.

## Supporting Information

S1 Fig Generation of the *Neurospora hac-1* knockout strain. A) Schematic representation of the *hac-1* (*NCU01856*) gene replacement event by a bialaphos-resistance (*bar*) cassette through homologous recombination. B) PCR was used to check for the presence of the *hac-1* gene in the WT strain and C) to evaluate the correct integration of the cassette used for *hac1* gene replacement in the homokaryon strain. D) Gel analysis of the PCR reactions depicted in B and C.
(DOC)

S2 Fig The promoters of *N*. *crassa grp78/bip* and *pdi* contain the *cis*-acting unfolded protein response element cUPRE-1. An alignment of known and putative HAC1 target promoters containing cUPRE-1 variants from different species is shown. The orange box marks the putative cUPRE-1 *cis* element. Conserved flanking nucleotides are shown in orange, using the known cUPRE-1 region from the yeast *KAR2* gene as the consensus sequence, as in [43]. For the *N*. *crassa* genes, the search for matches to the cUPRE-1 sequences was restricted to the first 1000 bp upstream of the start codon.
(DOC)

S3 Fig The Δ*hac-1* strain is unable to grow on cellulose as the sole carbon source. Conidia from WT (FGSC#988) and Δ*hac-1* strains were inoculated into flasks containing liquid Vogel’s medium (pH 5.8) with 2% (wt/vol) of the carbon source of interest (glucose, xylan or Avicel). Pictures were taken after 7 days of growth at 25°C in constant light conditions.
(DOC)

S1 Table Primers used in this study. When primers are described with both upper and lower case, the former denotes a region used for recombinational cloning and the latter, the specific target sequence.
(DOC)

S2 Table Putative Neurospora homologs of yeast genes involved in the unfolded protein response signaling pathway. (DOC)
