# Supplementary material for: The bZIP Transcription Factor HAC-1 Is Involved in the Unfolded Protein Response and Is Necessary for Growth on Cellulose in Neurospora crassa
Source: PLoS One. 2015 Jul 1;10(7):e0131415. doi: 10.1371/journal.pone.0131415 (PMC4488935; doi:10.1371/journal.pone.0131415)
Supplement: S1 Fig — A) Schematic representation of the hac-1 (NCU01856) gene replacement event by a bialaphos-resistance (bar) cassette through homologous recombination. B) PCR was used to check for the presence of the hac-1 gene in the WT strain and C) to evaluate the correct integration of the cassette used for hac1 gene replacement in the homokaryon strain. D) Gel analysis of the PCR reactions depicted in B and C. (DOC) [file pone.0131415.s001.doc]

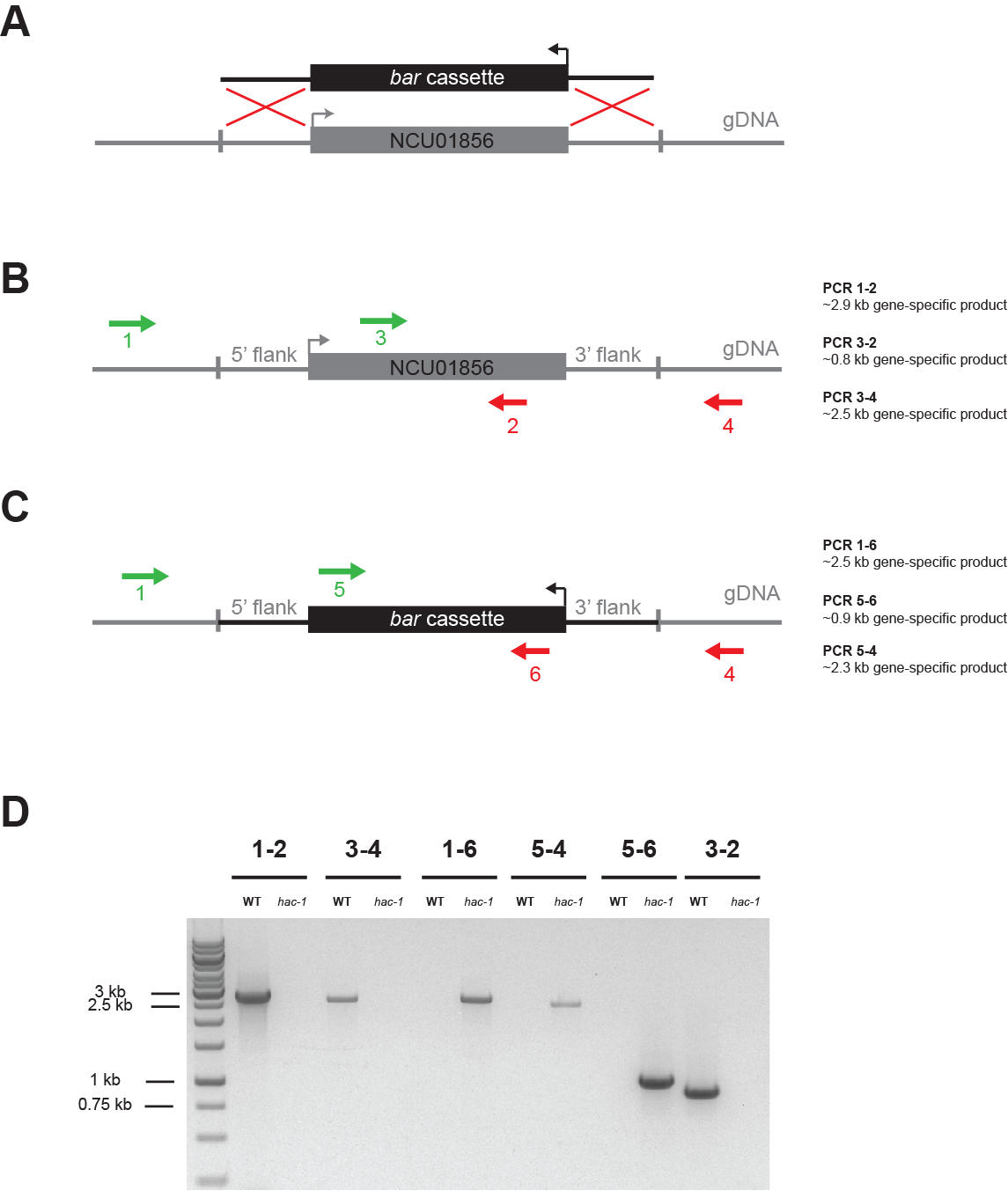


**Figure S1. Generation of the *Neurospora hac-1* knockout strain.** A) Schematic representation of the *hac-1* (*NCU01856*) gene replacement event by a bialaphos-resistance (*bar*) cassette through homologous recombination. B) PCR was used to check for the presence of the *hac-1* gene in the WT strain and C) to evaluate the correct integration of the cassette used for *hac1* gene replacement in the homokaryon strain. D) Gel analysis of the PCR reactions depicted in B and C.
