# Supplementary material for: The bZIP Transcription Factor HAC-1 Is Involved in the Unfolded Protein Response and Is Necessary for Growth on Cellulose in Neurospora crassa
Source: PLoS One. 2015 Jul 1;10(7):e0131415. doi: 10.1371/journal.pone.0131415 (PMC4488935; doi:10.1371/journal.pone.0131415)
Supplement: S2 Fig — An alignment of known and putative HAC1 target promoters containing cUPRE-1 variants from different species is shown. The orange box marks the putative cUPRE-1 cis element. Conserved flanking nucleotides are shown in orange, using the known cUPRE-1 region from the yeast KAR2 gene as the consensus sequence, as in [43]. For the N. crassa genes, the search for matches to the cUPRE-1 sequences was restricted to the first 1000 bp upstream of the start codon. (DOC) [file pone.0131415.s002.doc]

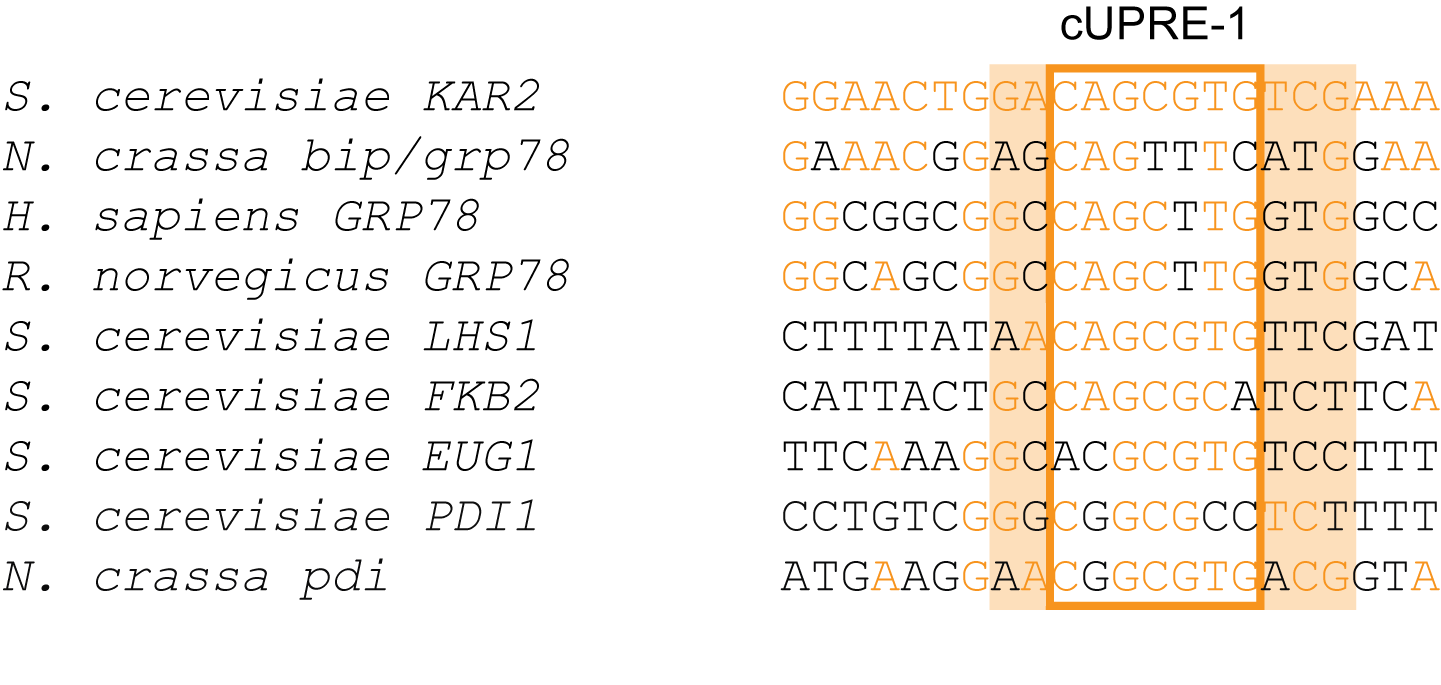


**Figure S2. The promoters of *N. crassa grp78/bip* and *pdi* contain the *cis*-acting unfolded protein response element cUPRE-1.** An alignment of known and putative HAC1 target promoters containing cUPRE-1 variants from different species is shown. The orange box marks the putative cUPRE-1 *cis* element. Conserved flanking nucleotides are shown in orange, using the known cUPRE-1 region from the yeast *KAR2* gene as the consensus sequence, as in [36]. For the *N. crassa* genes, the search for matches to the cUPRE-1 sequences was restricted to the first 1000 bp upstream of the start codon.
