# Supplementary material for: The bZIP Transcription Factor HAC-1 Is Involved in the Unfolded Protein Response and Is Necessary for Growth on Cellulose in Neurospora crassa
Source: PLoS One. 2015 Jul 1;10(7):e0131415. doi: 10.1371/journal.pone.0131415 (PMC4488935; doi:10.1371/journal.pone.0131415)
Supplement: S3 Fig — Conidia from WT (FGSC#988) and Δhac-1 strains were inoculated into flasks containing liquid Vogel’s medium (pH 5.8) with 2% (wt/vol) of the carbon source of interest (glucose, xylan or Avicel). Pictures were taken after 7 days of growth at 25°C in constant light conditions. (DOC) [file pone.0131415.s003.doc]

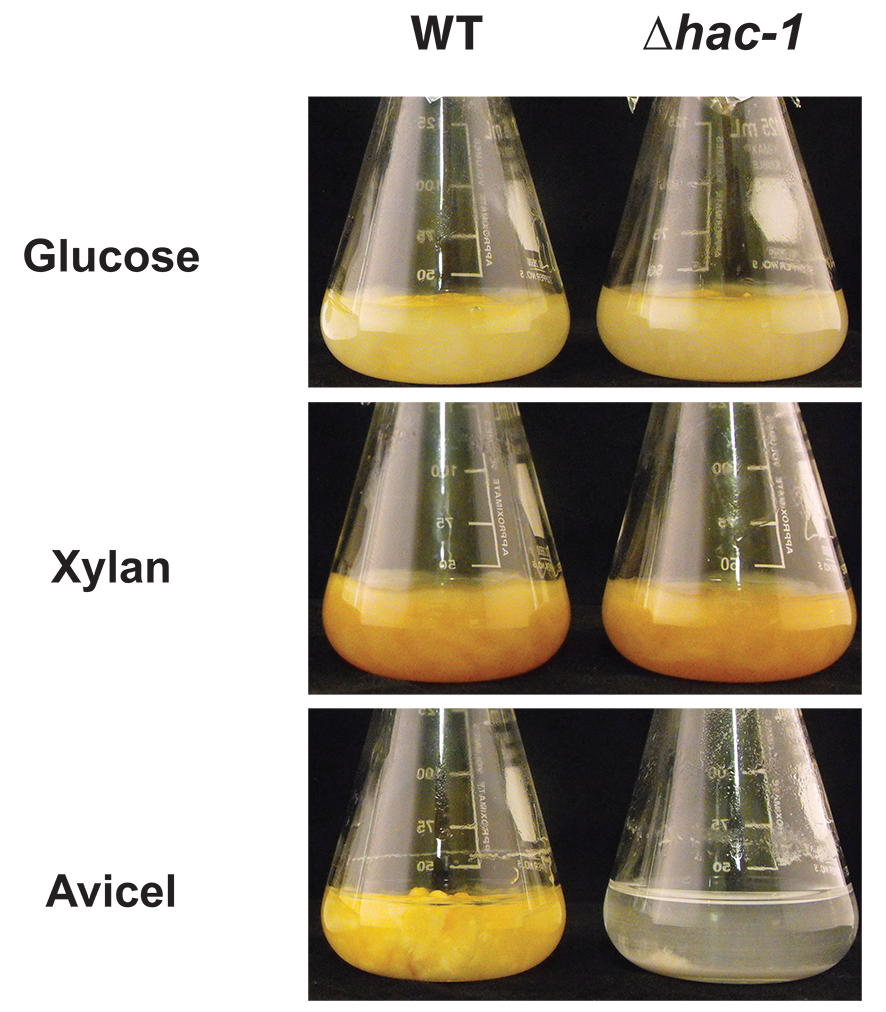


**Figure S3. The Δ*hac-1* strain is unable to grow on cellulose as the sole carbon source.** Conidia from WT (FGSC#988) and Δhac-1 strains were inoculated into flasks containing liquid Vogel’s medium (pH 5.8) with 2% (wt/vol) of the carbon source of interest (glucose, xylan or Avicel). Pictures were taken after 7 days of growth at 25° C in constant light conditions.
