# Supplementary material for: The bZIP Transcription Factor HAC-1 Is Involved in the Unfolded Protein Response and Is Necessary for Growth on Cellulose in Neurospora crassa
Source: PLoS One. 2015 Jul 1;10(7):e0131415. doi: 10.1371/journal.pone.0131415 (PMC4488935; doi:10.1371/journal.pone.0131415)
Supplement: S1 Table — When primers are described with both upper and lower case, the former denotes a region used for recombinational cloning and the latter, the specific target sequence. (DOC) [file pone.0131415.s004.doc]

**Table S1. Primers used in this study.** When primers are described with both upper and lower case, the former denotes a region used for recombinational cloning and the latter, the specific target sequence.

| **Primer name** | **Description** | **5'-3' Oligonucleotide sequence** |
| --- | --- | --- |
| 1 | KO genotyping | ggcgggaatagtctcatcaa |
| 2 | KO genotyping | gtcctctcccaaaacctgtg |
| 3 | KO genotyping | aaaacggtgttgctcctacg |
| 4 | KO genotyping | cgttctcacagagttgatcctc |
| 5 | KO genotyping | aagaccggcaacaggattc |
| 6 | KO genotyping | cttggctggagctagtggag |
| 5f | KO generation (nomenclature as in [92]) | GCGGATAACAATTTCACACAGGAAACAGCcctttagttgtacgaagatg |
| 5r | KO generation (nomenclature as in [92]) | ACATGTAATGCATAGTACCGAGAAACTAGTgatgggtatcggcgtccacg |
| 3f | KO generation (nomenclature as in [92]) | CCTTCAATATCATCTTCTGTCGAGTCTAGAaatcttgtatgacgtatatc |
| 3r | KO generation (nomenclature as in [92]) | GTAACGCCAGGGTTTTCCCAGTCACGACGcggacgtgctggagggcacc |
| Ignite-resistance cassette. Forward | KO generation | actagtttctcggtactatg |
| Ignite-resistance cassette. Reverse | KO generation | tctagactcgacagaagatg |
| Neurospora *hac-1* gene sequence. Forward | For Neurospora Δ*hac-1* complementation | GCGGATAACAATTTCACACAGGAAACAGCcctttagttgtacgaagatg |
| Neurospora *hac-1* gene sequence. Reverse | For Neurospora Δ*hac-1* complementation | CTGCAACAACCACCTCTCAAGGGCACCCACTtcatcaagacatccttcgac |
| Neurospora *actin* terminator sequence. Forward | For Neurospora Δ*hac-1* complementation | agtgggtgcccttgagaggtg |
| Neurospora *actin* terminator sequence. Reverse | For Neurospora Δ*hac-1* complementation | GCTCCTTCAATATCATCTTCTGTCTCCGACttggcaagatcaagaagccc |
| Hygromycin-resistance cassette. Forward | For Neurospora Δ*hac-1* complementation | gtcggagacagaagatgata |
| Hygromycin-resistance cassette. Reverse | For Neurospora Δ*hac-1* complementation | gttggagatttcagtaacgt |
| Neurospora *hac-1* 3’UTR sequence. Forward | For Neurospora Δ*hac-1* complementation | GATCCACTTAACGTTACTGAAATCTCCAACtttgaaaacacaatcttgta |
| Neurospora *hac-1* 3’UTR sequence. Reverse | For Neurospora Δ*hac-1* complementation | GTAACGCCAGGGTTTTCCCAGTCACGACGcggacgtgctggagggcacc |
| Neurospora *hac-1* CDS. Forward | For yeast Δ*hac1* complementation | AGCATACAATCAACTCCAAGCTTTGCAAAGatggattcctgggccgccca |
| Neurospora *hac-1* CDS. Reverse | For yeast Δ*hac1* complementation | AGGCTTACCTTCGAAGGGCCCTCTAGATCCgatacaacccaccgcaatac |
| *S. cerevisiae* *HAC1* CDS. Forward | For yeast Δ*hac1* complementation | AGCATACAATCAACTCCAAGCTTTGCAAAGatggaaatgactgattttg |
| *S. cerevisiae* *HAC1* CDS. Reverse | For yeast Δ*hac1* complementation | GGCTTACCTTCGAAGGGCCCTCTAGATCCtgaagtgatgaagaaatcattc |
| V5 tag. Forward | For yeast Δ*hac1* complementation | ggatctagagggcccttcg |
| V5 tag. Reverse | For yeast Δ*hac1* complementation | GGGTTTTTCAGTATCTACGATTCATAGATCtcaatggtgatggtgatgatg |
| Actin (*NCU04173*) Forward | RT-qPCR | ttaccgaggctcccatcaac |
| Actin (*NCU04173*) Reverse | RT-qPCR | ggcctggatggagacgtaga |
| Neurospora  *hac-1* (Total)  Forward  (Ref. 67) | RT-qPCR | aggaggatggcgagaagga |
| Neurospora  *hac-1* (Total)  Reverse  (Ref. 67) | RT-qPCR | cagtcgcagtggaggtagca |
| Neurospora  *hac-1* (Common for uninduced and induced versions)  Forward  (Ref. 67) | RT-qPCR | acctgttgcggatgctacct |
| Neurospora  *hac-1* (Uninduced)  Reverse  (Ref. 67) | RT-qPCR | acactgcaggtcatcacaca |
| Neurospora  *hac-1* (Induced)  Reverse  (Ref. 67) | RT-qPCR | cgaccgacactgcaggatg |
| Neurospora  *hac-1*  (Upstream of intron).  Forward | End-point  RT-PCR | gagaaggagcaatcgacgg |
| Neurospora  *hac-1*  (Downstream of intron).  Reverse | End-point  RT-PCR | ggagcagcagagagagagga |
| Neurospora  *hac-1*  (5’ UTR distal).  Forward | End-point  RT-PCR | tccttttcaccacacaaccaa |
| Neurospora  *hac-1*  (5’ UTR proximal).  Forward | End-point  RT-PCR | cagtgactgccacagctttc |
| Neurospora  *hac-1*  (5’ region).  Reverse | End-point  RT-PCR | accccacgacttcctcttct |
| *bip/grp78*  (*NCU03982*).  Forward | End-point  RT-PCR | gagattctcgtcaacgaccaa |
| *bip/grp78*  (*NCU03982*).  Reverse | End-point  RT-PCR | aagtcgtagacgatgatctga |
| *pdi*  (*NCU09223*).  Forward | End-point  RT-PCR | acgacattgttcttgctgaattc |
| *pdi*  (*NCU09223*).  Reverse | End-point  RT-PCR | cttctctttgagggtggtagc |
