# Supplementary material for: The bZIP Transcription Factor HAC-1 Is Involved in the Unfolded Protein Response and Is Necessary for Growth on Cellulose in Neurospora crassa
Source: PLoS One. 2015 Jul 1;10(7):e0131415. doi: 10.1371/journal.pone.0131415 (PMC4488935; doi:10.1371/journal.pone.0131415)
Supplement: S2 Table — (DOC) [file pone.0131415.s005.doc]

**Table S2. Putative Neurospora homologs of yeast genes involved in the unfolded protein response signaling pathway**

| *S. cerevisiae* gene | Neurospora putative homolog | E-value | Query Coverage |
| --- | --- | --- | --- |
| *HAC1 (YFL031W)* | *NCU01856* | 3e-17 | 23% |
| *IRE1 (YHR079C)* | *NCU02202* | 2e-138 | 88% |
| *Trl1/Rlg1 (YJL087C)* | *NCU04410* | 6e-163 | 95% |
| *PTC2* | *NCU04600* | 8e-98 | 66% |
